# Structural Health Monitoring Using Textile Reinforcement Structures with Integrated Optical Fiber Sensors

**DOI:** 10.3390/s17020345

**Published:** 2017-02-10

**Authors:** Kort Bremer, Frank Weigand, Yulong Zheng, Lourdes Shanika Alwis, Reinhard Helbig, Bernhard Roth

**Affiliations:** 1 Hannover Centre for Optical Technologies (HOT), Leibniz University Hannover, 30167 Hannover, Germany; Yulong.Zheng@hot.uni-hannover.de (Y.Z.); Bernhard.Roth@hot.uni-hannover.de (B.R.); 2 Saxon Textile Research Institute (STFI), 09125 Chemnitz, Germany; Frank.Weigand@stfi.de (F.W.); Reinhard.Helbig@stfi.de (R.H.); 3 School of Engineering and the Built Environment, Edinburgh Napier University, Edinburgh EH10 5DT, UK; L.Alwis@napier.ac.uk

**Keywords:** structural health monitoring, fiber optic sensor, crack detection, strain sensing, functionalized carbon structure

## Abstract

Optical fiber-based sensors “embedded” in functionalized carbon structures (FCSs) and textile net structures (TNSs) based on alkaline-resistant glass are introduced for the purpose of structural health monitoring (SHM) of concrete-based structures. The design aims to monitor common SHM parameters such as strain and cracks while at the same time acting as a structural strengthening mechanism. The sensor performances of the two systems are characterized in situ using Mach-Zehnder interferometric (MZI) and optical attenuation measurement techniques, respectively. For this purpose, different FCS samples were subjected to varying elongation using a tensile testing machine by carefully incrementing the applied force, and good correlation between the applied force and measured length change was observed. For crack detection, the functionalized TNSs were embedded into a concrete block which was then exposed to varying load using the three-point flexural test until destruction. Promising results were observed, identifying that the location of the crack can be determined using the conventional optical time domain reflectometry (OTDR) technique. The embedded sensors thus evaluated show the value of the dual achievement of the schemes proposed in obtaining strain/crack measurement while being utilized as strengthening agents as well.

## 1. Introduction

With the advancements in material sciences, the sensor research field is heading towards the implementation of an ultimate “nerves system” that can be utilized to sense various, if not all, physical, chemical and biological aspects not only of living beings but also of natural and/or man-made surroundings. This is evident from current trends towards the “smart city” concept, where the implementation of sensor systems to monitor the physical conditions of the civil structures of a city plays a vital role in addressing the economic benefits and ethical need for safe and sustainable infrastructures.

One main stakeholder of the said smart concept is the employment of appropriate structural health monitoring (SHM) schemes that can “sense” the physical conditions of structures to ensure their safe and efficient operation. Conventional techniques used for SHM are limited to electrical means, i.e., the use of strain gauges for strain measurement, for instance [1], where a pool of wires carrying a current not only poses a safety threat, but also is tedious to install and is not resource-efficient. Following the introduction and use of fiber optics during the telecommunications boom in the 1990s, both research and industry confidence had gathered momentum in the use of fiber optic technology not only for telecommunications purposes but also for sensing applications. Recent research by some of the authors [2] has also looked into the possibility of embedding sensors in smartphones, paving way for the wider application and use of fiber optic technology. In terms of SHM applications, fiber optic sensors (FOSs) have considerable advantages over their conventional electrical counterpart, i.e., being small in size, light weight, electrically passive and hence immune to electromagnetic interference. Since optical fibers are made of silica (glass), they are robust and can withstand harsh environments, i.e., FOSs can be used in high-temperature [3], radiation-hard [3,4,5] and corrosive environments [6] where electrical sensors cannot be used [7]. Due to the low light attenuation of optical glass fibers, they can be multiplexed and interrogated over several kilometers. The multiplexing and wider bandwidth capabilities render FOSs most suitable, therefore, when extracting data from a vast amount of sensing elements such as those required for SHM of larger areas, i.e., SHM of sewerage tunnels [7,8,9], dams [10] and bridges [11,12]. 

Conventional approaches to SHM using FOSs have been generally focused on grating-based sensors that are mounted to the structure afterwards, i.e., using epoxy for instance, so that effective measurement of physical parameters, such as strain [12,13], can be obtained. While these techniques are a step forward from electrical strain gauges, the need for specialist personnel to install them on the structure and the relative tediousness of having to glue them on the surface makes these schemes less attractive and flexible for civil engineers [14]. If the sensory elements can somehow be embedded with the strengthening mechanism of a structure, it would not only address the issue of having to install the sensors separately, but it would also pave way to a relatively more accurate system that would not require an intermediate transfer agent, e.g., the epoxy. Thus, it is of great interest to design a sensor system that could be “embedded” into the strengthening mechanism of a structure, providing the dual purposes of sensing and strengthening, saving a considerable amount of time and money.

Advances in material fabrication and processing engineering have rendered a step towards the possibility of “smart textiles” incorporating embedded sensors inherently in structures/textiles in order to not only mitigate the risk of failure due to an overload or unwanted inhomogeneity resulting from the fabrication process [15], but also for the identification, assessment and location detection of physical parameters, i.e., such as strain, experienced by the composite containing the sensors [16,17]. The integration or embedding of the sensors, in general, is achieved by sewing or warp-knitting the sensors into the host structure [18]. The interrogation of the sensors is then achieved by the utilization of fiber Bragg grating (FBG) [13,15,19] and different distributed fiber optic sensor systems [18].

**Figure 1:**
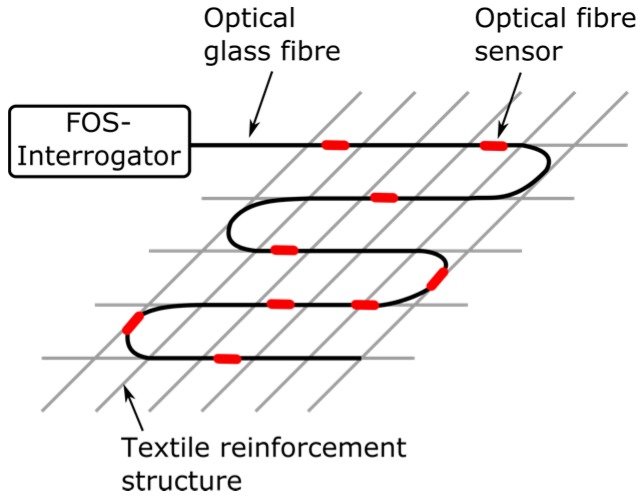
Sketch of a textile reinforcement structure that is functionalized with fiber optic sensors in order to monitor important SHM parameters. The textile reinforcement structures are designed so that they can act as both sensing and strengthening mechanism.

In light of the discussion above, the work presented here introduces the design and evaluation of a tailored functionalized carbon structure (FCS), used for strengthening, with integrated optical fiber sensors to extract important SHM parameters (e.g., strain), and a fiber optic crack sensor based on a textile net structure (TNS) to be used for SHM. The work proposed here explicitly targets crack and strain detection in concrete-based composite structures, specifically in line with and as a progression of former work on SHM of sewerage tunnels [8,9] of some of the authors. The “embroidery” principle of operation of both proposed fiber optic sensor concepts is shown in Figure 1. Thus, the new design of optical fiber sensors acts simultaneously as the reinforcement as well as the SHM system, not only making it more practical and hence easier for civil engineers to employ, but also saving time and money while ensuring more accuracy compared to conventional FOSs (which rely on a transfer agent).

## 2. Fiber Optic Crack Sensor

For concrete-based civil engineering structures, the formation of cracks and the resulting moisture ingress is critical since it will continuously deteriorate the health of the structure. In order to determine the failure of the structure, early detection of the crack formation is essential. Therefore, in this work, a fiber optic crack sensor is developed which is based on a TNS consisting of alkaline-resistant glass and an integrated optical single-mode fiber. As illustrated in Figure 1, the textile net structure is designed to transfer elongation due to cracks of the concrete structures to the optical fiber, and hence the TNS will break the integrated optical fiber. Since the optical fiber is pre-strained during the stitching on top of the TNS, the optical fiber will break at smaller crack sizes compared to the alkaline-resistant glass. Furthermore, the position of the crack and, hence, the failure position of the concrete structure can be identified by using optical time domain reflectometry (OTDR), for instance, which is established in the field.

### 2.1. Crack Sensor Material and Fabrication

**Figure 2:**
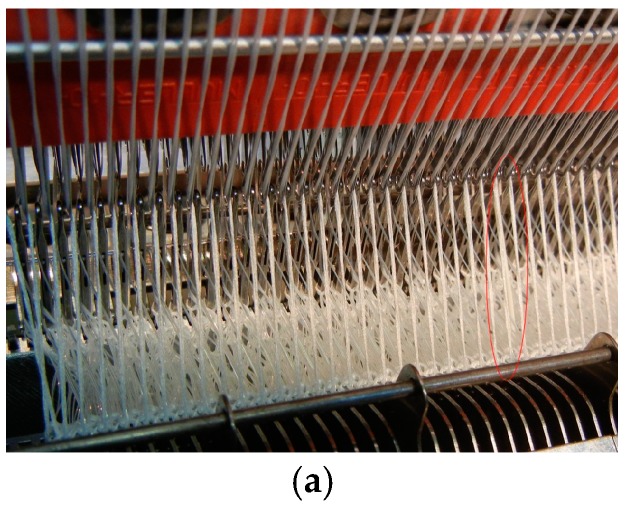

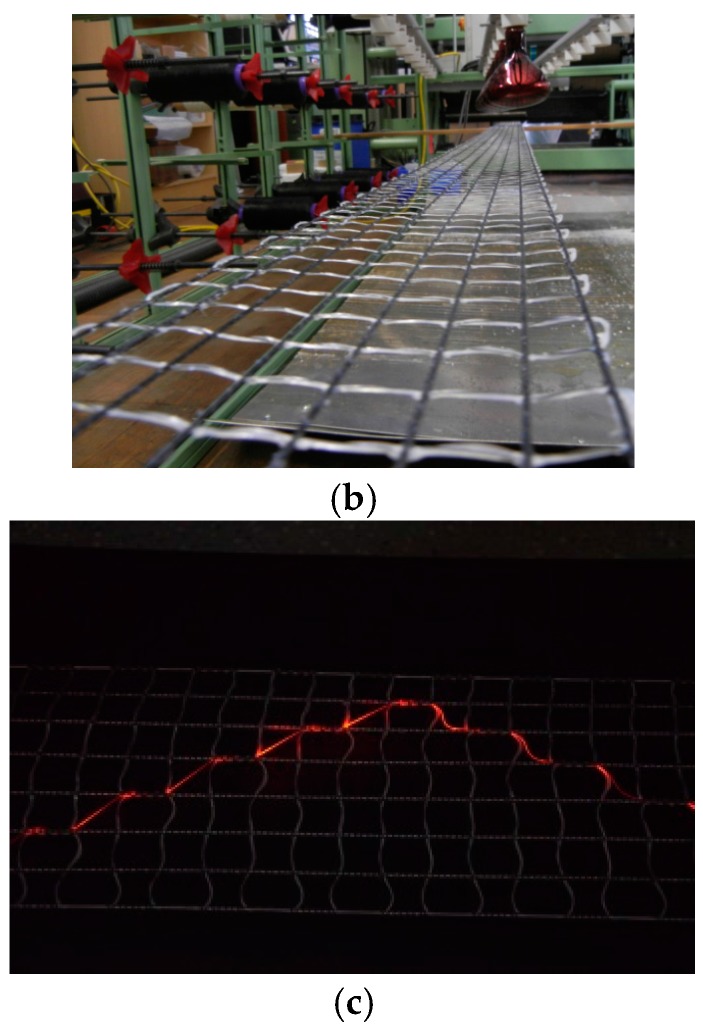
Wrap knitting machine that can simultaneously process alkaline-resistant glass and optical glass fibers (**a**) and, hence, which was applied to fabricate the fiber optic crack sensor (**b**,**c**).

For the integration of optical fibers in the TNS, an appropriate reel-to-reel knitting technique has been developed at the Saxon Textile Research Institute (STFI) in Chemnitz, Germany. The TNS is a biaxial grid with a spacing of 20 mm that has been fabricated in-house at the STFI using a wrap knitting machine and a 2400 tex alkaline-resistant glass multifilament in 0° and 90° directions and a polypropylene multifilament as a thread with 44 tex. The optical glass fiber is integrated instantaneously during the knitting process of the TNS. At the end, the whole textile-based sensing structure is stabilized by applying a copolymer coating. The developed stitching process provides a strong bonding between the optical fiber sensor and the textile net structure while introducing only low, light bend losses into the optical fiber applied. To achieve low bending losses, the best result was obtained with the bend-optimized optical single-mode fiber from Corning (Corning^®^ ClearCurve^®^ single-mode fiber with acrylate coating; after integration the measured attenuation was <0.2 dB/m). In Figure 2a, the wrap knitting machine that was applied to fabricate the textile net structure with integrated optical fiber sensors is illustrated. Moreover, a fabricated fiber optic crack sensor is shown in Figure 2b,c. In Figure 2c, the light of a red laser was coupled into the integrated optical fiber to visualize the resulting path of the optical sensor.

### 2.2. Experimental Set-Up and Evaluation of the Fiber Optic Crack Sensor

**Figure 3:**
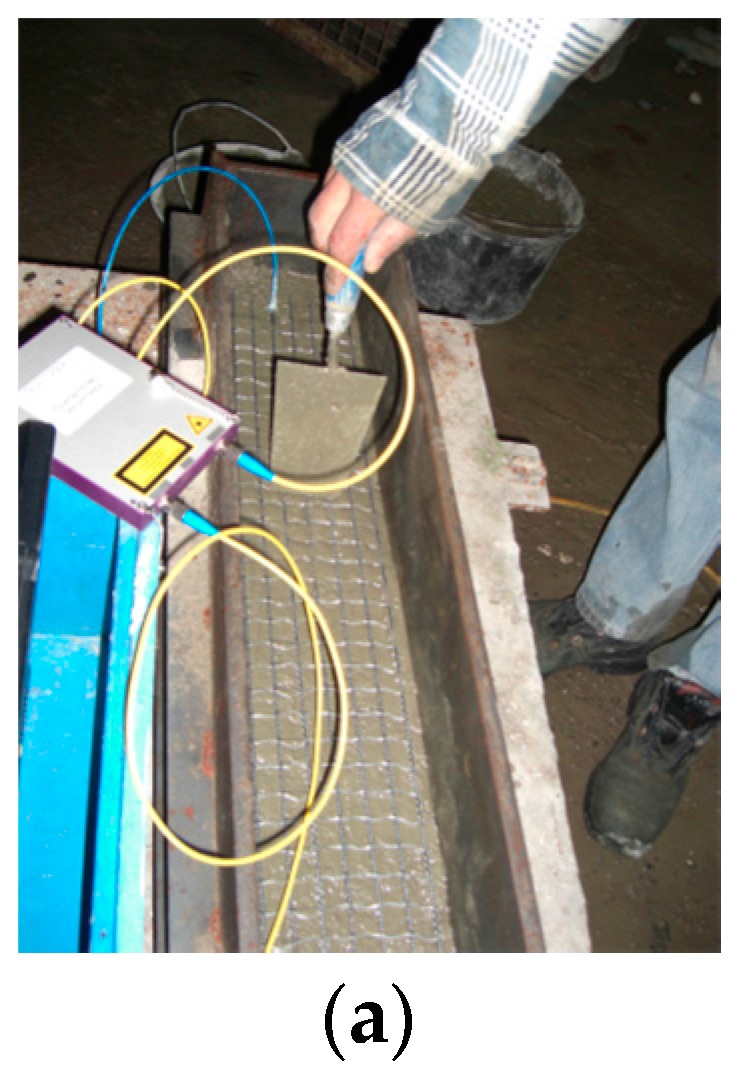

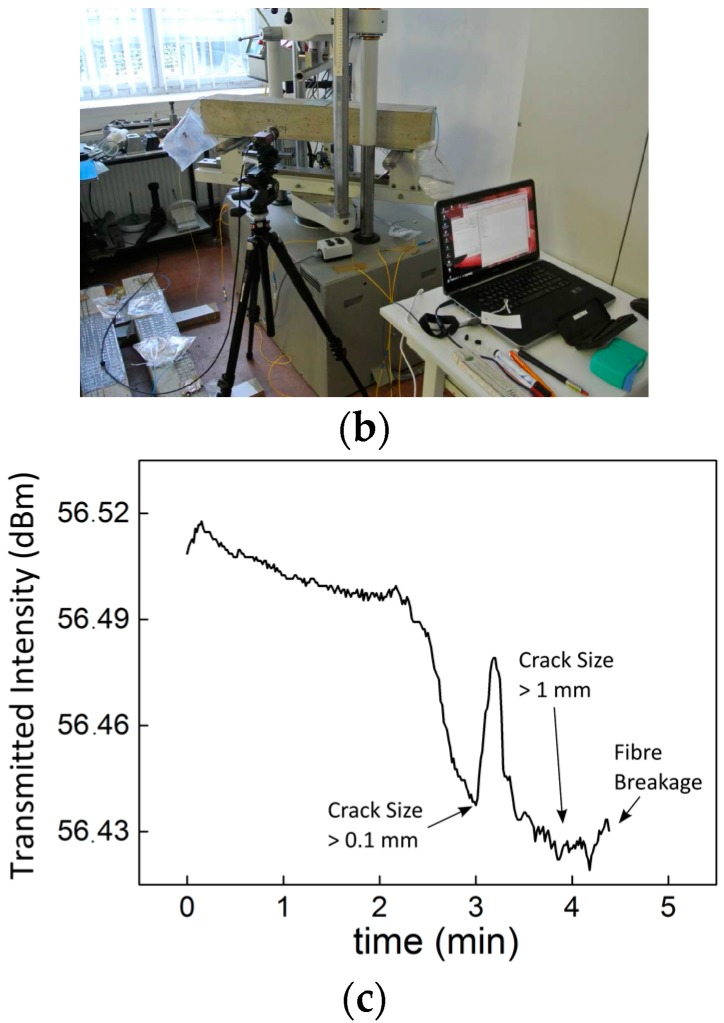
Embedding the sensor in a concrete block (**a**) and experimental set-up (**b**) for the evaluation of the fiber optic crack sensor performance (**c**).

The performance of the embedded crack sensor was evaluated by embedding the TNS containing the sensor unit into a concrete block with dimensions of 100 cm × 15 cm × 15 cm and cracking the fabricated concrete element after curing at a defined location by applying a load using the established three-point flexural test. By simultaneously monitoring the attenuation of the optical fiber and measuring the crack size, the sensor’s performance was characterized. The fabrication of the concrete block with an embedded textile-based fiber optic crack sensor is shown in Figure 3a. For the experiment, the textile-based sensor was also used as a reinforcement of the concrete block. The light attenuation of the optical fiber was measured during the experiment using a dB-meter from FiboTec and the obtained crack size was determined using a digital camera, as shown in Figure 3b. In Figure 3c, the measured light attenuation of the sensor at different crack sizes is illustrated. At a crack size of 1.4 mm, the optical fiber inside the textile net structure broke and indicated the failure of the concrete block. Moreover, from initializing the crack formation up to the fiber breakage, the attenuation of the transmitted light was less than 0.09 dB, as illustrated in Figure 3c. Therefore, combined with the OTDR interrogation technique, the TNS-based fiber optic crack sensor can be applied for distributed SHM. However, compared to other distributed fiber optic sensor techniques, such as stimulated Brillouin scattering, the fiber optic crack sensor cannot be applied for quantitative SHM measurement, i.e., online evaluation of the structural health, since no correlation between the crack size and light attenuation was obtained.

## 3. Carbon Reinforcement Structures with Integrated Optical Fiber Strain Sensors

Developments in material sciences and nano-technology, especially in polymer engineering, have rendered a variety of sensing possibilities, one recent advancement being the utilization of carbon fibers and carbon nanotubes not only for various sensing purposes [20] but also as strengthening agents, i.e., the utilization of carbon fiber reinforcement polymer (CFRP) for strengthening structures such as railway bridges [12]. In addition, the relatively small size and large flexibility of optical fibers opens the possibility of embedding optical fibers in carbon reinforcement structures directly during the manufacturing process [21]. This will not only enable carbon reinforcement structures to additionally monitor measurements such as strain and cracks, but will also enhance the protection of the fragile sensing elements, i.e., especially in the case of using fiber Bragg gratings [12]. In addition, optical fibers embedded in a grid-like composite would be able to perform multi-point sensing, paving the way to create a profile of the target measurement, i.e., a strain profile of a bridge, for instance.

This approach also provides a promising potential for utilizing FCSs to be tailor-made for concrete composite applications, both as a strengthening and sensing agent, and thus addressing two important aspects simultaneously. The FCS manufacturing process could therefore be “designed” in a manner, to target a specific shape for instance, that could be utilized to create the “embedded” sensing/strengthening mechanism mentioned above, as is also evident from the work presented here. 

### 3.1. Functionalized Reinforcement Sensor Design, Material and Fabrication

**Figure 4:**
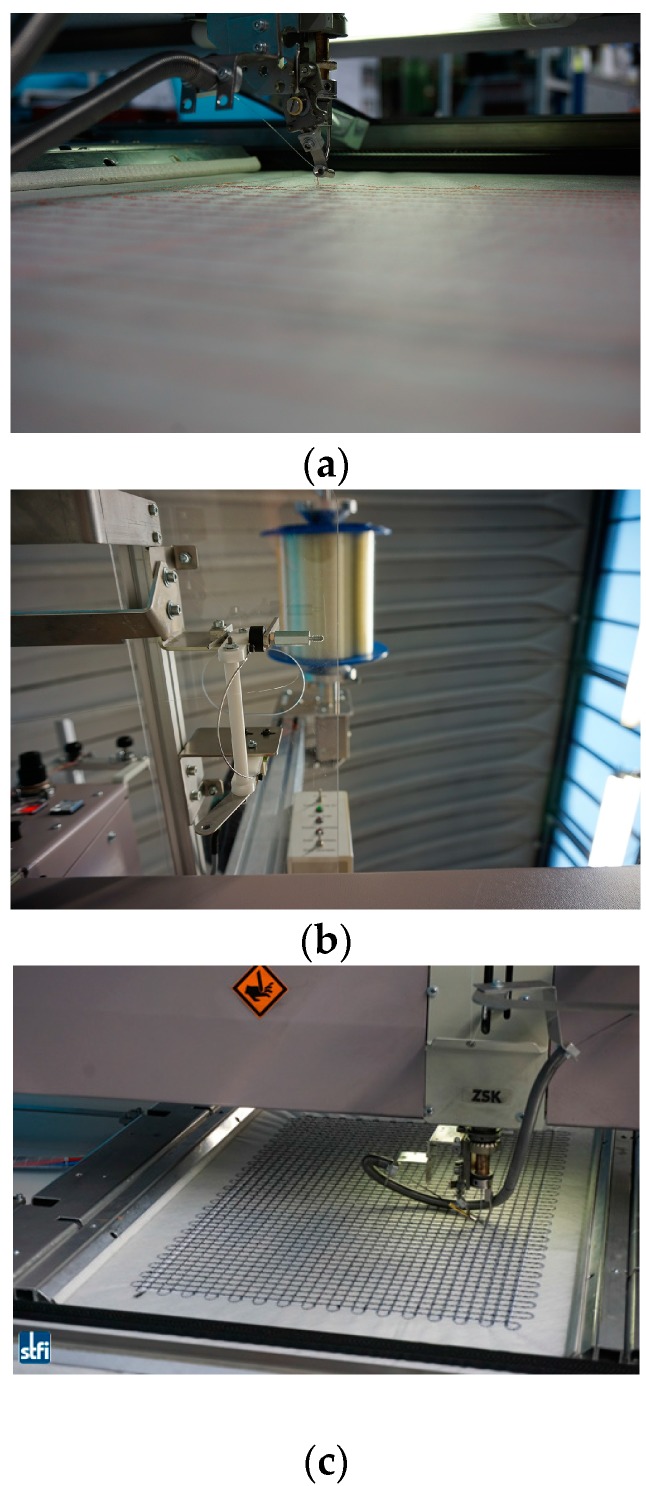
Embroidery machine that was modified in order to simultaneously process carbon filaments and optical fibers. The modification included the development of an appropriate embroidery needle (**a**) and a mechanism to auto-track the optical fiber during fabrication of the FCS (**b**) to process the carbon filaments and optical glass fiber simultaneously on a nonwoven substrate made of PVA (**c**).

The embroidery technique that is employed for the FCS fabrication was also developed at STFI. This was achieved through the manufacturing of the functionalized carbon structure (FCS) with the optical fiber simultaneously “woven” to the FCS. To do so, carbon fibers and optical fiber sensors were processed to produce grid-like elements. The advantage of this technique is the possibility of fabricating a tailored carbon structure, i.e., several layers of carbon filaments with up to 50 k filament fibers (corresponding to 3200 tex), as well as different shapes of grid structures, depending on the targeted application and purpose. Figure 4a,b depict the modified embroidery machine that was used for simultaneous processing of the carbon fiber filaments and optical fibers used for the work presented here.

The fabrication of the “interwoven” carbon/optical-fiber strands was followed by the realization of a tailored textile-based FCS. To do so, first the optical fiber (Corning^®^ ClearCurve^®^ single-mode fiber with acrylate coating) and carbon filaments were embroidered on a polyvinyl alcohol (PVA) nonwoven substrate. Several layers of carbon fibers were incorporated subsequently on the PVA nonwoven substrate in order to obtain the required tailored grid-like structure. Upon completion of the fabrication process, the nonwoven substrate was then removed by dissolving the PVA in hot water (approximately 50 °C). The fabricated functionalized grid reinforcement structure embroidered on the PVA substrate is shown in Figure 4c.

### 3.2. Experimental Set-Up to Evaluate FCS

The careful characterization of the sensor’s performance is of high importance, given the fact that the integrated optical fiber is intended to be utilized for the SHM of carbon-reinforced elements. The characterization, in particular, should include the evaluation of force transfer from the carbon structure to the optical fiber as will be experienced in the target application, and any hysteresis and/or drift of the sensor.

When applied in the laboratory environment, interferometric techniques have the advantage of providing a relatively cost-efficient (no special fibers and interrogation systems are required) and accurate approach to characterize the performance of optical fiber sensors. Therefore, for an initial proof-of-principle experiment on the performance of the FCS, a Mach-Zehnder interferometric (MZI) set-up was applied. The set-up was designed so that the changes that are introduced due to the applied force on the optical fiber (which was incorporated in the FCS) can be detected, and hence the resulting force transfer between the carbon reinforcement structure and the optical fiber can be evaluated. 

**Figure 5:**
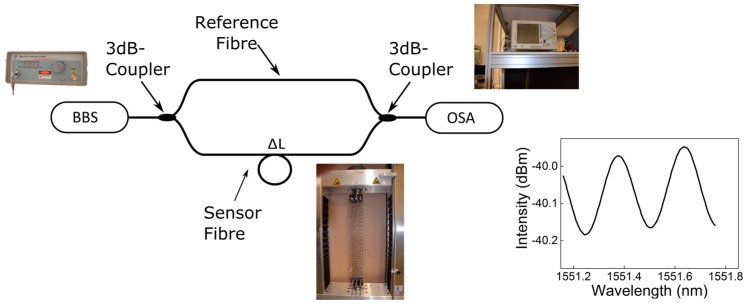
Developed fiber optical MZI to evaluate and characterize the force transfer between the carbon structure and the optical fiber of the FCS.

The fiber-optic MZ interferometer that was used for this purpose is shown in Figure 5. The set-up consists of a broadband light source (BBS, Opto-Link C-Band ASE, Hong Kong, China), an optical spectrum analyzer (OSA, ANDO AQ6317B, Tokyo, Japan), two 3 dB couplers and single-mode fiber optic reference/sensor arms. Any force-related change of the sensor arm causes a phase difference (Δφ) between the light (of wavelength *λ*) traveling in both fiber arms, resulting in an interference pattern change, as displayed in the subset of Figure 5. The obtained phase difference can be used to calculate the length change of the MZI.

### 3.3. Evaluation of Sensor Performance of the FCS 

To fully evaluate the fiber optic strain sensor’s performance, two aspects are highlighted: first, the evaluation of the force transfer between the carbon filaments and the optical fiber; second, the evaluation of the “sensors embedded structure” to assess whether this structure can be applied for sensing purposes as well. To assess the two aspects, the evaluation of the fiber optic strain sensor’s performance was thus divided into two sections. In order to only characterize the force transfer between the carbon filament and the optical fiber, only one-dimensional (1D) functionalized carbon structures were initially investigated. However, for structures that have to provide both strengthening and sensing mechanisms, two-dimensional (2D) FCSs are required. Therefore, subsequently, a two-dimensional FCS was characterized and its sensitivity to longitudinal and normal forces was evaluated.

**Figure 6:**
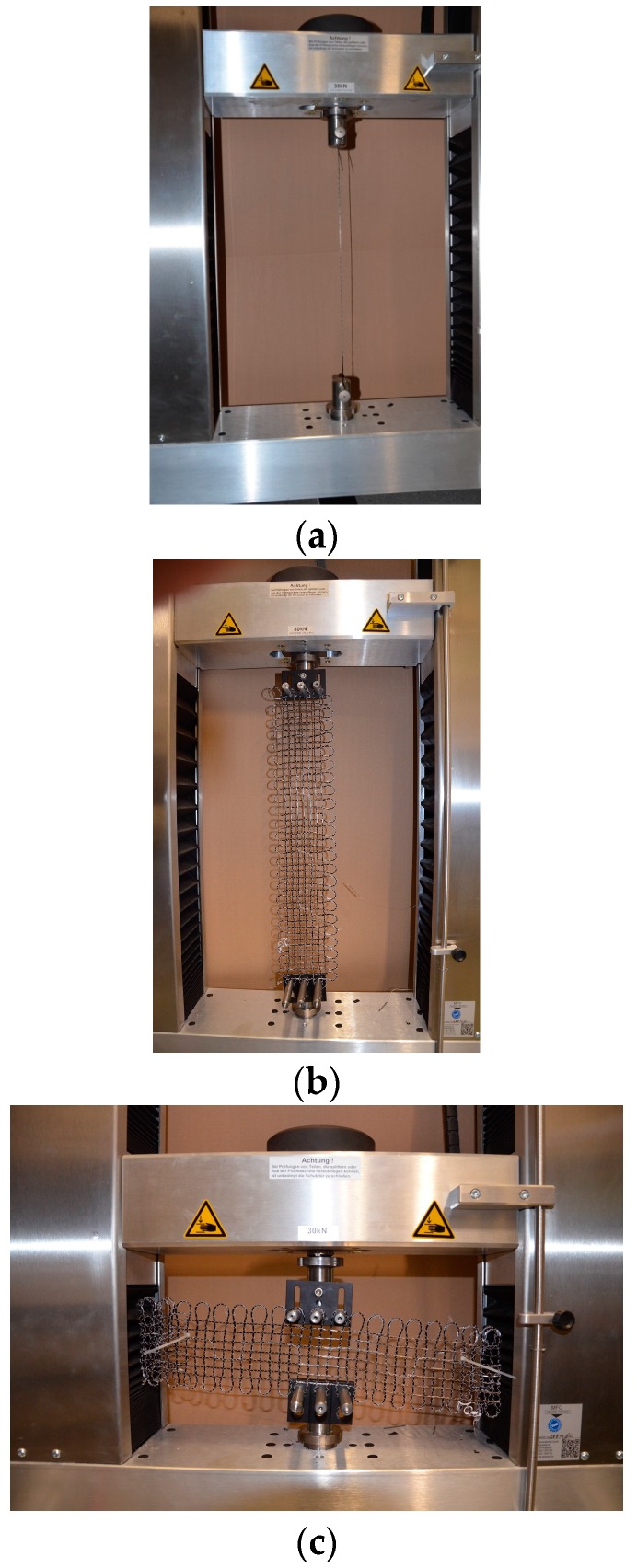
Functionalized 1D and 2D FCSs mounted to the tensile testing machine MFC T3000 in order to evaluate the force transfer between the carbon filament and the optical fiber (**a**) as well as the sensitivity to longitudinal (**b**) and normal (**c**) forces.

In Figure 6, the experimental set-up to evaluate the sensor’s performance for the functionalized carbon structures based on a tensile testing machine, MFC T3000, is illustrated. In Figure 6a, the force transfer between the carbon filament and the optical fiber is investigated. Following this, the force transfer of longitudinal and normal forces of the 2D-FCS is explored, as shown in Figure 6b,c.

For the force transfer evaluation between the carbon filament and the optical fiber, a one-dimensional carbon skein 30 cm in length and a 1600 tex carbon filament that was integrated with a Corning^®^ ClearCurve^®^ single-mode fiber with acrylate coating was used. The functionalized carbon skein was embroidered first on the PVA nonwoven substrate and then cut into 30-cm-long samples following the removal of the PVA (water-dissolved). This was then followed by the splicing of the integrated optical fiber to the above MZ interferometer and the sensor’s performance was characterized using the tensile testing machine MFC T3000. The force transfer between the textile carbon structure and optical fiber was evaluated by mounting only the carbon structure to the tensile testing machine and subjecting the structure to applied force in the range from 0 to 350 N. Furthermore, before the measurement started, the 1D FCS was pre-strained at a force of 5 N.

Following the evaluation of the 1D structures, the sensor’s performance for the 2D functionalized carbon structures was evaluated. The 2D structures were fabricated using the same fabrication technique described above. The 2D structures with 1600 tex were 520 × 110 mm^2^, and a grid spacing of 10 mm, and the optical fiber positioned vertically in the middle of the grid were utilized. Upon integration, the integrated optical fiber was spliced to the MZ interferometer and the sensor’s performance was characterized (Figure 6b,c). In order to avoid breaking the carbon filament and the optical fiber during the experiments, the maximum length change in the horizontal and vertical directions was ≤1%. 

**Figure 7:**
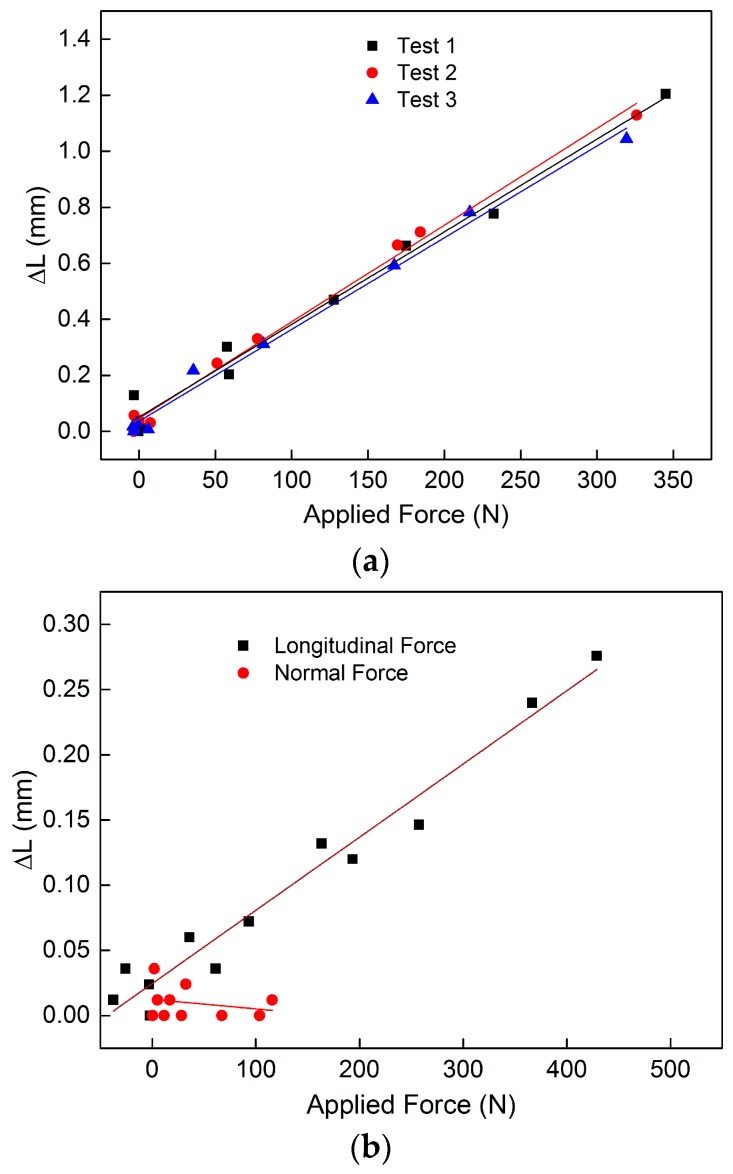
Response of the 1D FCS sample (**a**) to applied force as well as the response of the 2D FCS to applied longitudinal and normal forces (**b**).

The force transfer of the 1D FCS thus evaluated is shown in Figure 7a. Three subsequent tests were performed for the 1D FCS in order to characterize the hysteresis and drift. As is obvious from Figure 7a, a good correlation between the applied force and the measured length change Δ*L* can be seen, with a linearity of 0.0033 mm/N (±1.4%) for forces up to 350 N. Furthermore, a relatively small standard derivation of 0.042 mm/N was obtained for the one-dimensional FCS. The measured sensor performance of the 2D-FCS when longitudinal and normal forces were applied is shown in Figure 7a,b. Before the measurement, the 2D FCS was pre-strained at a force of 100 N. The 2D FCS shows a good correlation with the applied longitudinal forces (5.6 × 10^−4^ mm/N) and no cross-sensitivity to normal forces, as can be seen from Figure 7b. The reduced sensitivity to the applied force compared to the 1D case can be attributed to the increased cross-section of the 2D FCS. Hence, depending on the tex number and the geometric shape of the FCS, the sensitivity and measurement range can be optimized. Furthermore, a relatively small hysteresis and drift to the applied force were observed (not shown), which is consistent with the results achieved for the 1D FCS. The characterized FCSs that are shown in Figure 7a,b are based on acrylate-coated optical glass fibers. Currently, the influence of different fiber coatings on the sensor performance of the FCS is being investigated. 

## 4. Discussion

The two different functionalized reinforcement structures with integrated optical fiber sensors for SHM applications were successfully realized using textile stitching techniques. The techniques ensured careful integration of the optical fibers, which is essential for realizing reliable distributed sensor systems. The fiber optic crack sensor provides a simple means to detect crack-related failures of concrete structures and responses to cracks ≥1.4 mm. Since the transmitted light intensity of the fiber optic crack sensor shows no correlation to the crack size, as illustrated in Figure 3c, only the breakage of the fiber can be used as an indication of the status of the structural health. Therefore, once the sensor is activated, i.e., the textile structure transfers the crack and breaks the optical fiber, the TNS-based crack sensor has to be renewed. Consequently, the fiber optic crack sensor system is only suitable for qualitative monitoring applications, i.e., the sensor can only provide information about whether a structure is still operating or has failed, and for situations where, in case of a failure, the whole concrete element has to be renewed, which is, e.g., relevant for concrete sewerage pipes [8,9]. For sewerage tunnels, the most common inspection technique is currently based on a remote video camera–based system. Because of the complex nature of this inspection process, it is usually only applied at regular intervals. Therefore, the fiber optic crack sensor provides a simple and cost-efficient approach for the continuous monitoring of the structural health of concrete-based sewerage tunnels.

In contrast, the carbon reinforcement structures with integrated optical fiber strain sensors can be applied for quantitative SHM applications, and thus can be used to determine the status of a structure and hence are able to predict the event of a failure. Combined with a MZI interrogation technique, the developed FCSs show a linear response to applied longitudinal forces with a relatively small hysteresis and drift (0.0033 mm/N (±1.4%)), with a standard derivation of 0.042 mm/N for 1D FCS. Furthermore, since the cross-sensitivity to normal forces can be neglected, the direction of the applied force can be determined depending on the position of the FCSs relative to the measured object. Therefore, when the MZI interrogation technique is replaced for in-field use by FBGs or stimulated Brillouin scattering, the developed FCSs provide a novel means for distributed SHM. Currently, the operation of the FCS in combination with FBG sensors is being explored when two-dimensional structures are embedded in concrete elements.

## 5. Conclusions

The work presented here highlights the design, realization, and evaluation of fiber optic sensors embedded in carbon and textile reinforcement structures for the application of concrete composite structures, respectively. The fiber optic crack sensor is based on light attenuation measurement, where the optical fiber was stitched on the surface of a textile net structure which was then placed in a concrete block that was subjected to crack formation. The sensor responded well to cracks resulting from three-point flexural tests (until destruction) and, due to the breakage of the optical fiber, the crack locations could be identified using the OTDR technique. On the other hand, the functionalized carbon structures were based on an optical fiber interwoven with carbon fiber filaments (simultaneously during manufacturing) which were then exposed to applied force and interrogated using a MZI set-up. The functionalized carbon reinforcement samples indicated a strong and reliable correlation between the applied force and measured elongation with relative small hysteresis and drift. Both reported fiber optic sensor approaches are based on single-mode optical fiber with acrylate coating. However, depending on the application, multi-mode (MM) fiber can also be applied. The strength of the sensor approaches proposed lies in the fact that sensing and strengthening can be simultaneously achieved, saving considerable cost and time that would otherwise be required. Furthermore, the use of appropriate and effective technology, i.e., OTDR, FBGs or stimulated Brillouin scattering, also plays a key role in achieving timely, reliable and reproducible status information for a broad range of structures. The evaluation of both sensor schemes has provided promising results in the approach applied for opening up new research avenues in multi-purpose sensing mechanisms for SHM applications.
